# Diffusive Formation of Au/Ag Alloy Nanoparticles of Governed Composition in Glass

**DOI:** 10.3390/nano12234202

**Published:** 2022-11-26

**Authors:** Ekaterina Babich, Igor Reduto, Andrey Lipovskii

**Affiliations:** 1 Laboratory of Nanophotonics, Alferov University, Khlopina 8/3, 194021 Saint Petersburg, Russia; 2 Institute of Physics and Mechanics, Peter the Great St. Petersburg Polytechnic University, Polytechnicheskaya 29, 195251 Saint Petersburg, Russia

**Keywords:** alloy nanoparticles, gold, silver, glass, ion-exchange

## Abstract

For the first time we show that the introduction of silver ions in the glass containing gold nanoparticles (NPs) and additional heat treatment of the glass in the air lead to the formation of Au/Ag alloy NPs. The proposed approach makes it possible to position localized surface plasmon resonance of the NPs by selecting the heat treatment temperature, which determines the silver proportion in the alloy NPs. This allows for expanding customizability of NPs for applications in surface-enhanced Raman scattering spectroscopy, catalysis and biochemistry. Developed technique benefits from the presence of silver in the glass in ionic form, which prevents the oxidation of silver and provides stable preparation of Au/Ag alloy NPs.

## 1. Introduction

The interaction of light with free electrons of metal nanoparticles (NPs) results in the excitation of localized surface plasmon resonance (LSPR) in NPs. When excited, NPs behave similar to resonators and exhibit enhanced absorption and scattering of light [1]. This allows using metal NPs in various areas, such as medicine, ecology, and even economy and art. For example, NPs have been used as nanosources of heat for cancer treatment [2], sensitive elements of biochemical detectors of SARS-CoV-2 [3], components of solar cells for light trapping [4] and anticounterfeiting tags for product identification [5]. Most of these applications use Au and Ag NPs due to their stability and significantly higher quality factor (Q-factor) of LSPR compared to NPs of other metals [6]. The versatile applicability of NPs is ensured by the possibility of tuning their optical characteristics. This can be conducted by changing the size, shape or composition of NPs. While changing the size and shape of NPs allows the LSPR to be positioned in a limited spectral range, changing the composition of NPs allows adjusting the LSPR over a wide spectral range. For example, spherical Ag and Au NPs of different sizes cover 400–450 nm [7] and 520–570 nm [8] spectral ranges, respectively. Alternatively, the LSPR of Au/Ag alloy NPs can be positioned from 400 to 570 nm [9]. Proper positioning of the LSPR of alloy NPs provides an increase in the enhancement of Raman scattering compared to monometallic NPs [10,11] and the sensitivity of colorimetric detection of biomarkers [12]. Moreover, control over the composition of alloy NPs makes it possible to use them as photothermal agents in NIR spectral range [13], antibacterial agents with low cytotoxicity [14], and highly catalytic agents [15]. For this reason, Au/Ag alloy NPs have recently received considerable attention.

By now, several methods have been proposed for forming Au/Ag alloy NPs.

It is demonstrated that “wet” co-reduction of HAuCl_4_ and AgNO_3_ in a reducing agent [16,17,18] and seed-mediated growth [19] allows the synthesis of Au/Ag alloy NPs of given composition in a large amount. However, these methods lead to the preparation of NPs in the form of colloids. Such colloids are prone to aggregation both in solution and upon adsorption on a substrate [20,21]. To avoid this one should additionally either stabilize [22] or cover [23] the NPs for use in catalysis or biotechnology or before deposition on a substrate for use in surface-enhanced Raman scattering spectroscopy (SERS).

More convenient “dry”, method to form stable alloy NPs directly on a substrate is co-depositing and depercolation of ultra-thin Au and Ag films. The latter can be provided by heat treatment [24,25] or laser irradiation [26,27]. Immediately after formation, such NPs are ready for use in SERS. Unfortunately, the composition and morphology of the resulting alloy NPs are extremely sensitive to the annealing/irradiation regime and film thicknesses [28,29]. In addition, Ag films tend to oxidize [30] which also affects resulting NPs.

In this work, we demonstrate a new “dry” method that allows the stable formation of Au/Ag alloy NPs directly on a glass substrate, avoiding the use of Ag film. It consists of heat treatment of Au film deposited on a sodium-containing glass with the formation of Au NPs. This is followed by the introduction of Ag^+^ ions into the glass via silver-to-sodium ion exchange. The final heat treatment of the glass containing both Au NPs and Ag^+^ ions leads to the reduction of Ag^+^ to neutral silver accompanied by the diffusive formation of Au/Ag alloy NPs. The proportion of Ag in the alloy NPs depends on temperature, which makes it possible to govern LSPR in the spectral range of 500–570 nm. The latter is essential for the use of these NPs, e.g., in SERS, since it allows one to adjust the range of maximal Raman enhancement for given light sources and analytes.

## 2. Materials and Methods

### 2.1. Formation of Nanoparticles

To obtain Au NPs we deposited gold film on the surface of soda-lime glass slides (Agar Scientific Ltd., Essex, UK) using an RF magnetron sputter coater (Q300T T Plus, Quorum, Laughton, UK). The nominal thickness of the film was 5 nm (mass equivalent controlled by calibrated quartz crystal monitor). Then the specimens were annealed in air at 650 °C for 30 min. Annealing of the thinner (3 nm) film led to the formation of an extremely small number of Au NPs, while the thicker (10 nm) Au film remained percolated.

To introduce Ag^+^ ions in the glass containing Au NPs, the ion-exchange replacement of sodium ions in glass with silver ions from AgNO_3_-NaNO_3_ melt [31] was used. The proportion of silver in the melt, the temperature and the duration of ion exchange determine the maximum concentration and depth of penetration of Ag^+^ ions in the glass, respectively. We used the melt containing 5 wt.% of AgNO_3_. The melt was heated to 325 °C, and the glass containing Au NPs was immersed in the melt for 20 min. The glass was cleaned in an ultrasonic bath of acetone:isopropanol (50:50) solution before and after the immersion. The estimated maximum concentration and penetration depth of Ag^+^ ions amounted to ~10 wt.% and 10 µm, respectively [32].

Heat treatment in the air of the glass containing Ag^+^ ions and Au NPs led to the reduction of Ag^+^ to Ag^0^, and the number of reduced ions depended on the treatment temperature. The reduction is followed by both clustering of silver with the formation of Ag NPs and alloying of silver and gold with the formation of Au/Ag NPs. The treatment temperature was chosen in the range of 250–600 °C to control the proportion of Ag in the alloy NPs; the processing time was 15 min. For comparison, we also treated glasses containing either only Ag^+^ ions or Au NPs.

### 2.2. Characterization

The morphology of formed NPs was characterized using a scanning electron microscope (SEM, 1550 Gemini, Leo, Oberkochen, Germany) and atomic-force microscope (AFM, Dimension-3100, Veeco, Plainview, NY, USA). The SEM and AFM images were analyzed using ImageJ public domain software (NIH, Bethesda, MD, USA) and Gwyddion free software (gwyddion.net (accessed on 25 November 2022), Brno, Czech Republic), respectively.

Extinction (absorbance) spectra of the glass with NPs were measured with a UV-VIS spectrophotometer (Specord 50, Analytik Jena, Jena, Germany). In the spectra, we separated the contribution of NPs formed on the glass surface from the contribution of NPs grown in the near-surface layer of the glass. To do this, the spectra were measured before and after the removal of surface NPs. The NPs were removed by cleaning the glass surface with cotton buds soaked in petroleum ether. Thus, the difference between the spectra measured before and after NPs removal (differential spectra) corresponded to surface NPs, while the spectra obtained after NP removal (bulk spectra) corresponded to near-surface NPs.

To determine the thickness of the glass layer containing NPs the glass was etched with monitoring the extinction spectra. We used either a solution of HF (5 µL):NH_4_F (5 g):H_2_O (40 g) (“strong solution”), providing a fast etching of ∼75 nm/min, or a solution of NH_4_F (1 g):H_2_O (100 g) (“weak solution”), which provides slow etching at a rate of ∼4 nm/min. When the glass became transparent, the step between etched and unetched areas was measured using a laser scanning confocal microscope (LEXT OLS5000, Olympus, Tokyo, Japan).

## 3. Results and Discussion

### 3.1. Formation of Au Nanoparticles

**Figure 1:**
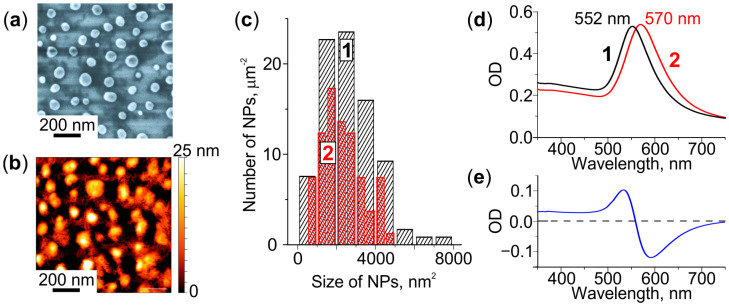
Characterization of the glass with Au NPs. (**a**) SEM and (**b**) AFM images. (**c**) NPs size distributions before (1) and after (2) cleaning of the glass surface. (**d**) Extinction spectra of the as-received (1) and cleaned (2) glass, (**e**) corresponding differential spectrum.

The SEM and AFM images presented in Figure 1a,b, respectively, show that heat treatment of the Au film deposited on the glass leads to the formation of NPs on the glass surface. The NPs are sparsely distributed (about 80 NPs per µm^2^) and have an elliptical shape. Their average major and minor axes are ~77 and ~67 nm, respectively. The average height is ~22 nm. Most of the NPs remain on the glass even after cleaning it in an ultrasonic bath or with the cotton buds. The comparison of particle size (area in the lateral plane) distributions obtained from the SEM images of the glass before and after cleaning indicates that only a small number of larger NPs can be removed (see Figure 1c). However, after 1 min etching of the glass in the “weak” solution (etching rate of 4 nm/min) all NPs can be wiped out from the surface. We assume that NPs formed on the glass surface as the result of depercolation of the thin Au film [33], are partially immersed in the glass at the temperature above the glass transition temperature (Tg = 565 °C) [34]. Thus, the glass-NPs interface is located 4 nm below the initial surface of the glass.

We measured the extinction spectra before and after cleaning the glass surface. Figure 1d shows that the removal of larger NPs from the surface leads to a long-wavelength shift of the spectrum by 18 nm. The NPs remaining on the glass demonstrate LSPR at ∼570 nm. Thus, the differential spectrum (Figure 1e) is negative in the 560–750 nm spectral range.

### 3.2. Formation of Au/Ag NPs

To form Au/Ag alloy NPs we introduced Ag^+^ ions in the glasses containing Au NPs and heat-treated the glasses in the air at 250–600 °C. For comparison, we also heat-treated glasses containing either only Ag^+^ ions or Au NPs.

#### 3.2.1. Heat Treatment of the Glass Containing Ag^+^ Ions

**Figure 2:**
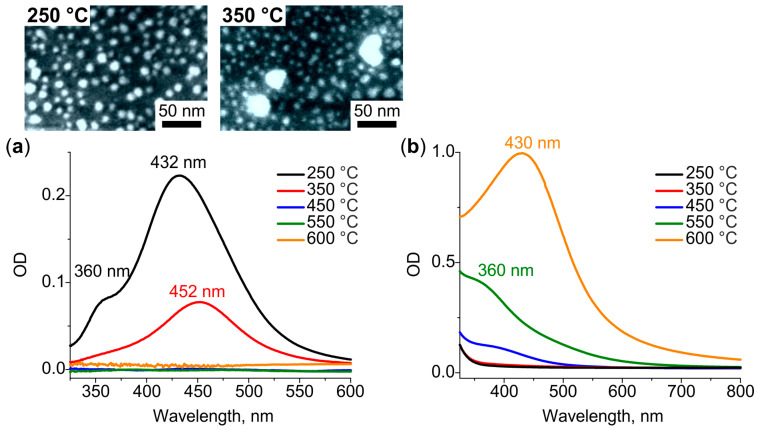
(**a**) Differential and (**b**) bulk spectra corresponded to surface and bulk Ag NPs, respectively, formed in a glass containing Ag^+^ ions after heat treatment in air at 250–600 °C. Inset: SEM images of surface Ag NPs formed at 250 °C and 350 °C.

Figure 2a shows that heat treatment of the glass containing Ag^+^ ions at 250 °C leads to the emergence of two distinct resonances in the differential spectrum. One of the resonances is at 360 nm and the other is at 432 nm. When the temperature of the treatment increases to 350 °C the short-wavelength resonance vanishes from the spectrum, while long-wavelength resonance experiences the redshift by 20 nm, and its intensity decreases by a factor of 2. The SEM images of both (250 °C and 350 °C) heat-treated specimens (see inset in Figure 2a) demonstrate the presence of circularly shaped NPs ~10 nm in diameter. Larger NPs (~30 nm) could also be observed on the surface of the glass treated at 350 °C. For the temperatures of heat treatment above 350 °C, the differential spectrum has no resonances, while the bulk spectrum does (see Figure 2b). The resonance in the bulk spectrum shifts from 360 nm to 430 nm, and its intensity increases by tenfold as the treatment temperature rises from 450 °C to 600 °C. Note, the SEM images of the glasses treated at ≥450 °C do not show any NPs on the surface. We interpret the transformation of the spectra and SEM images as follows.

In the course of the heat treatment, Ag^+^ ions are reduced to Ag^0^ atoms by water vapours diffused in glass from the air [35,36], and by electrons released by the glass network (via breaking of the bond of non-bridging oxygen atoms of the glass with silver ions) [37,38]. At a low temperature of heat treatment, 250 °C, the depth of water vapors diffusion in soda-lime glass is about 750 nm [39,40]. Thus, silver ions are reduced to atoms only in a thin subsurface layer of glass. The atoms diffuse towards the strongest sink, that is the glass surface, and aggregate in Ag nanoclusters and small NPs (out-diffusion) [41]. Partially oxidized Ag nanoclusters and NPs demonstrate LSPRs exactly at 360 and 435 nm, respectively [42]. Increasing the heat treatment temperature by 100 °C (to 350 °C) increases the depth of diffusion of water vapors in the glass. Thus, the number of reduced subsurface silver atoms also increases. The vanishing of the resonance corresponded to Ag nanoclusters from the differential spectrum of the glass treated at 350 °C may indicate the aggregation of nanoclusters with the formation of NPs. The shift of Ag NPs LSPR towards longer wavelength should be associated with the enlargement of NPs [43], which is seen in the corresponding SEM image (inset in Figure 2a). We assume, the shift could also be associated with the stronger oxidation of Ag NPs, which can explain the dumping of resonance intensity [44].

The following increase in the temperature of the heat treatment (≥450 °C) results in deeper diffusion of water vapors and reduction of silver ions everywhere in the ion-exchanged region of the glass via the decomposition of silver oxide [38]. Both the absence of Ag NPs on the glass surface demonstrated by SEM images and the appearance of LSPR resonance only in the bulk spectra indicate the formation of Ag atomic clusters in the glass bulk. These clusters should be a stronger sink than the glass surface, and one can expect the behavior of Ag clustering similar to one caused by reactive hydrogen diffusion [45]. According to the bulk spectra, first, Ag nanoclusters appear in glass at 450 °C, then their number increases at 550 °C, and finally, they aggregate with the formation of Ag NPs at 600 °C. The experimentally estimated thickness of the region containing Ag clusters/NPs is ~5, 11 and 31 µm for the glass treated at 450, 550 and 600 °C, respectively. Given the thickness of the ion-exchanged glass, the region is about 10 µm, almost half of the ions are reduced in glass at 450 °C, and all ions at 550 and 600 °C. Moreover, heat treatment at 600 °C shifts silver atoms concentration front deeper in the glass.

#### 3.2.2. Heat Treatment of the Glass Containing Au NPs

**Figure 3:**
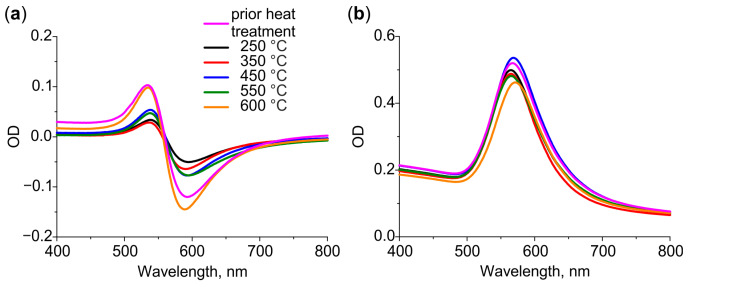
(**a**) Differential and (**b**) bulk spectra of glasses containing Au NPs after heat treatment in air at 250–600 °C.

Differential and bulk spectra of the glasses with Au NPs after heat treatment at different temperatures are presented in Figure 3a,b, respectively. The glasses were cleaned prior to the treatment. As we have demonstrated in Section 3.1, the cleaning results in the removal of larger NPs from the surface. This induces the shift of the LSPR towards a longer wavelength and results in the appearance of negative values in the differential spectrum. Thus, the spectra of heat-treated glasses reflect low-rate out-diffusion of gold atoms: the intensity of the bulk spectrum decreases, while the intensity of the differential spectrum increases with the temperature of heat treatment. The maximum change in the intensity is observed for 600 °C: the bulk spectrum drops by 15% of the initial value, while the differential spectrum becomes the same as it was before the heat treatment. We assume, this is because of the increase in solubility of gold in glass at a temperature exceeding T_g_.

It is important that the spectral position of Au NPs LSPR in bulk spectra does not change with the temperature of heat treatment. This indicates that the size and shape of Au NPs do not change significantly. Indeed, analysis of SEM images of the glass surface before and after heat treatment at 600 °C does not show significant changes in particle size distribution (see Figure S1 in Supplementary Material).

#### 3.2.3. Heat Treatment of the Glass Containing Both Ag^+^ Ions and Au NPs

**Figure 4:**
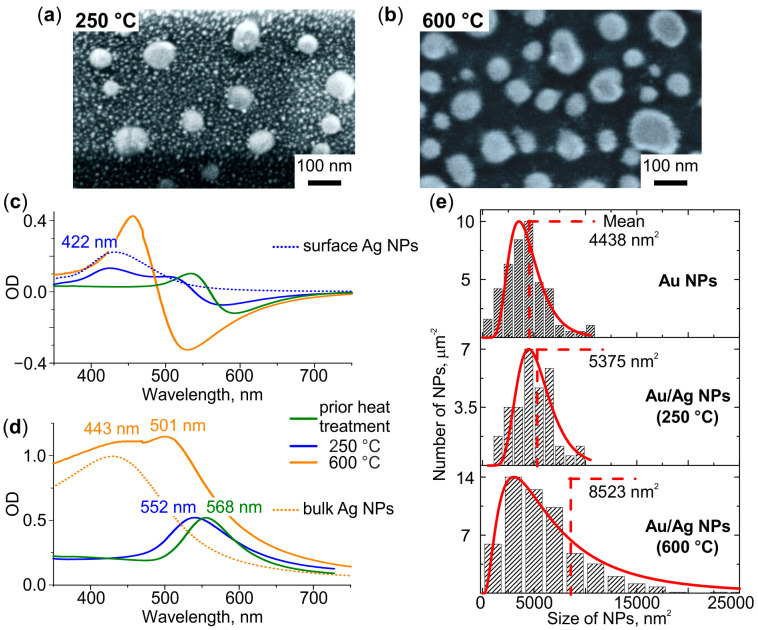
SEM images of the glasses containing Au NPs and Ag^+^ ions after heat treatment in air at (**a**) 250 °C and (**b**) 600 °C. (**c**) Differential and (**d**) bulk spectra of the treated glasses. The dotted spectra correspond to glasses containing Ag NPs on the surface (blue) or in the bulk (orange). (**e**) NPs size distributions in glasses containing Au and Au/Ag NPs (formed at a different temperature, denoted), and corresponding log-normal approximations (red). Mean sizes of NPs are denoted.

Figure 4a,b demonstrate SEM images of the glasses after the sequential formation of Au NPs, introducing Ag^+^ ions, and heat treatment of these specimens at 250 °C and 600 °C, respectively. It can be seen that the treatment at 250 °C leads to the formation of two types of NPs: small circularly shaped densely-spaced and large elliptically shaped sparsely-distributed ones. The treatment at 600 °C leads to the formation of the only type of NPs—large elliptically shaped and sparsely-distributed. To reveal the nature of the formed NPs we analyzed their morphology and optical properties, and compared these with the characteristics of Ag and Au NPs formed after the same heat treatment in the glasses which contained either Ag^+^ ions or Au NPs.

The average diameter of small circularly shaped NPs formed after heat treatment at 250 °C is ~10 nm. The corresponding differential spectrum demonstrates additional (compared to the spectrum of the glass before introducing Ag^+^) resonance at 422 nm (see Figure 4c). Both the size and the spectral position of the resonance correspond to Ag NPs (compare with Figure 2a). Thus, the small NPs are Ag NPs formed on the surface of the glass. The average major and minor axes of large elliptically shaped NPs formed after the heat treatment at 250 °C are ~82 and ~70 nm, respectively. These NPs are slightly larger than Au NPs (compare with Figure 1a). The corresponding bulk spectrum shows a blue shift of their resonance relative to the LSPR of Au NPs (see Figure 4d). It was shown [46,47,48,49,50] that the presence of only one resonance band, which shifts to the blue side with an increase in the fraction of Ag in the Au/Ag alloy, confirms the formation of NPs in the Au/Ag alloy. Thus, the observed blue-shift evidences alloying Au with Ag.

The heat treatment at 600 °C leads to the formation of one type of NPs, which are larger than the ones formed after the treatment at 250 °C. The average major and minor axis of the NPs are ~93 and ~74 nm, respectively. The corresponding differential spectrum experiences blue-shift relatively to the spectrum of the glass treated at 250 °C, but no additional resonances related to Ag NPs can be seen (see in Figure 4c). Thus, Ag NPs are not formed on the glass surface, and the only NPs visible in the SEM image are Au/Ag ones. The bulk spectrum demonstrates two resonances at 443 and 501 nm. It is seen that the short-wavelength resonance corresponds to the spectrum of the glass containing Ag NPs in the bulk (dotted line in Figure 4d). Thus, Ag NPs form in glass bulk. The long-wavelength resonance corresponded to Au/Ag NPs in the bulk spectrum is blue-shifted in relatively to the LSPR of Au NPs and Au/Ag NPs formed at 250 °C (see Figure 4d). It is known that an enlargement of NPs causes a redshift of their LSPR [51]. However, we registered the blue shift of the LSPR of the enlarged NPs. This indicates an increase in the proportion of Ag in Au/Ag NPs with increasing heat treatment temperature.

Comparison of the average size of Au/Ag NPs with the initial size of Au NPs allows for estimating the proportion of Ag in the alloy NPs. In particular, the lateral sizes of Au/Ag NPs formed after heat treatment at 250 °C and 600 °C are, on average, *F* = 1.1 and *F* = 1.4 times larger than the ones of Au NPs, respectively (see Figure 4e). The height of Au/Ag NPs increased in proportion to lateral sizes (see Figure S2 in Supplementary Material). Assuming that (i) the mass of gold in NPs, *m_Au_*, did not change in the course of the heat treatment, and (ii) the density of alloy is linearly related to its composition, the mass of silver in Au/Ag NPs, *m_Ag_*, can be evaluated using the equation:

(1) $$1+\frac{D_{Au}\cdot m_{Ag}}{D_{Ag}\cdot m_{Au}}=F^{3}$$

where *D_Au_* = 19.3 g/cm^3^ and *D_Ag_* = 10.5 g/cm^3^ are densities of Au and Ag, respectively. The calculated *m_Ag_* equals to 0.18∙*m_Au_* and 0.9∙*m_Au_* for Au/Ag NPs formed after heat treatment at 250 °C and 600 °C, respectively. Thus, the proportion of Ag in Au/Ag NPs increases from ~15% to ~48% with the increase in the temperature of heat treatment.

**Figure 5:**
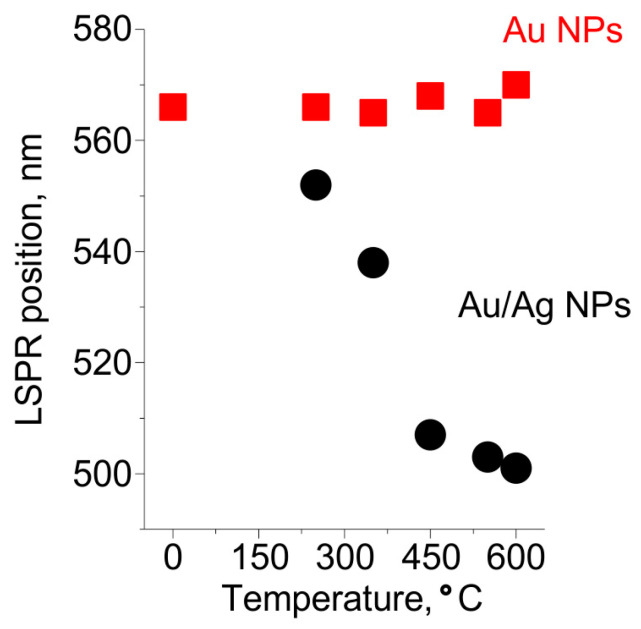
The dependencies of spectral positions of Au and Au/Ag NPs resonances on the temperature of heat treatment.

To reveal the mechanism of Au/Ag NPs formation we studied the dependencies of the spectral positions of Au NPs and Au/Ag NPs LSPRs on the temperature of heat treatment in 250–600 °C range. The dependencies are presented in Figure 5. One can see that Au NPs LSPR is not influenced by the treatment, while the LSPR of Au/Ag NPs gradually shifts from 550 nm towards a lower wavelength with the increase of the temperature of the heat treatment from 250 °C to 450 °C. When the glass is treated at the higher temperature the LSPR remains at the same wavelength, ~500 nm.

We interpret the transformation of the Au/Ag NPs resonance as follows.

The mobility of silver in glass is much higher than that of gold [52], and the silver self-diffusion coefficient in gold is high. According to [53], silver can diffuse in gold at the depth of ~750 nm upon annealing for 15 min at 600 °C. Therefore, alloying of silver with gold has to occur faster than the out-diffusion of gold to the glass surface. Thus, Au NPs formed in the glass prior to introducing Ag^+^ ions are a sink for silver atoms. The ratio of silver fluxes to Au NPs and to other sinks (the glass surface or Ag NPs) depends on the position of the front of atomic silver concentration in the glass. Thus, the front position determines the proportion of Ag in Au/Ag NPs. This position is governed by the penetration depth of water vapors, which reduce Ag^+^ ions to atomic silver. The vapors penetrate in glass up to a micrometer at temperatures below 350 °C. By this, only silver ions near the surface are reduced to atoms, which out-diffusion leads to the formation of small Ag NPs on the glass surface and Au/Ag alloy NPs with low Ag fraction. These alloy NPs provide a shift of LSPR by less than 30 nm relative to the position of the LSPR of Au NPs. Increasing of the heat treatment temperature to 450 °C leads to a deeper (up to 5 µm as estimated from the experimental data presented in Section 3.2.1) penetration of water vapors in the glass. This results in an essential increase in the number of reduced silver atoms in the glass. Consequently, the proportion of Ag in Au/Ag alloy NPs increases. Indeed, the observed shift of Au/Ag NPs LSPR in this case is about 60 nm. Further increase of the temperature of heat treatment from 450 °C to 600 °C does not lead to a shift of Au/Ag NPs LSPR. This evidences that the proportion of Ag in Au/Ag NPs does not change. We believe, this is because of the increased solubility of gold in the glass and an aggregation of silver in the glass bulk (see Figure 2b) at these temperatures.

## 4. Conclusions

It has been shown for the first time that the introduction of Ag^+^ ions in the glass containing Au NPs and additional heat treatment of the glass in the air lead to the formation of NPs of the Au/Ag alloy. This method makes it possible to position the LSPR of NPs in the spectral range of 500–570 nm by selecting the heat treatment temperature, which determines the Ag proportion in the Au/Ag NP alloy. The Ag proportion in the NPs can be changed from 15 to 48%. Developed technique benefits from the presence of silver in the glass in ionic form. The latter prevents the oxidation of silver, in contrast to known methods that use the co-deposition of gold and silver films on a substrate and, thus, provides stable preparation Au/Ag alloy NPs. Obtained NPs could find promising use in SERS providing an increase in sensitivity via tuning position of the LSPR to SERS excitation wavelength or characteristic Raman band of the analyte.

## Supplementary Materials

The following supporting information can be downloaded at: https://www.mdpi.com/article/10.3390/nano12234202/s1, Figure S1: Au NPs size distributions before (black) and after (blue) heat treatment in air at 600 °C; Figure S2: AFM profiles of the glass surface (from left to right) with Au film as-deposited on glass, the Au film after annealing at 650 °C, and after the annealing and following introducing Ag^+^ ions in glass and additional heat treatment at 600 °C.
