# Supplementary material for: Diffusive Formation of Au/Ag Alloy Nanoparticles of Governed Composition in Glass
Source: Nanomaterials (Basel). 2022 Nov 26;12(23):4202. doi: 10.3390/nano12234202 (PMC9738725; doi:10.3390/nano12234202)
Supplement: Supplementary file 1 [file nanomaterials-12-04202-s001.zip › nanomaterials-2050727-supplementary.pdf]

SUPPLEMENTARY MATERIAL

Diffusive Formation of Au/Ag Alloy Nanoparticles of Governed Composition in Glass

Ekaterina Babich [1], Igor Reduto [1] and Andrey Lipovskii [1,2,*]

[1] Laboratory of Nanophotonics, Alferov University, Khlopina 8/3, 194021 Saint Petersburg, Russia
[2] Institute of Physics and Mechanics, Peter the Great St. Petersburg Polytechnic University, Polytechnicheskaya 29, 195251 Saint Petersburg, Russia
* Correspondence: lipovsky@spbau.ru

Figure S1 shows particle size (area in the lateral plane) distributions obtained from SEM images of the glass with Au nanoparticles (NPs) before and after heat treatment in air at 600 °C. One can see that the treatment weakly influence the size of NPs.

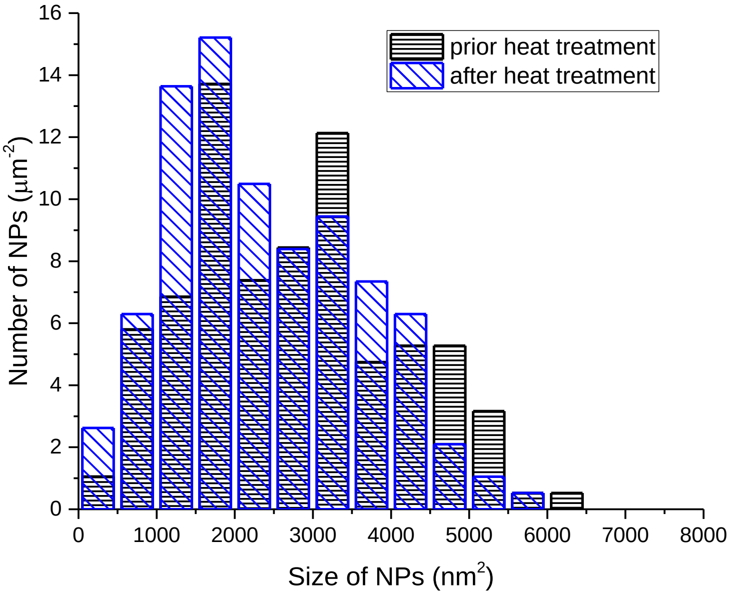

Figure S1. Au NPs size distributions before (black) and after (blue) heat treatment in air at 600 °C.

Figure S2 shows measured with Atomic Force Microscope (AFM) profiles illustrating the evolution of the of Au film, which was deposited on glass, upon annealing in air at 650°C and following introducing Ag^+ ions in glass and heat treatment in air at 600°C. One can see that the thickness of the deposited film is about 5 nm, and surface roughness is ~1 nm. The annealing leads to depercolation of the film and formation of Au NPs with the average height ~ 22 nm. The introducing of Ag^+ ions in the glass containing Au NPs and additional heat treatment lead to formation of Au/Ag alloy NPs with the average height ~31 nm.

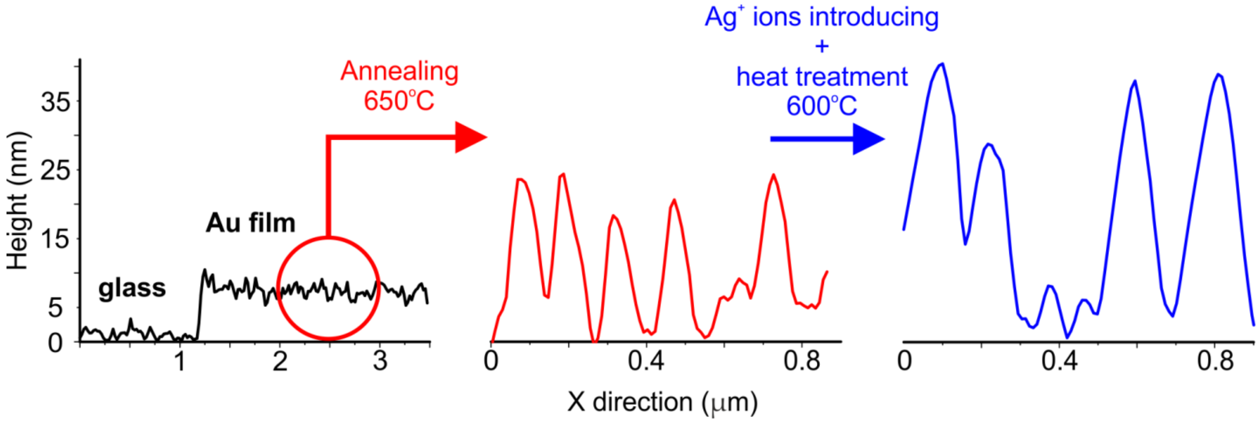

Figure S2. AFM profiles of the glass surface (from left to right) with Au film as-deposited on glass, the Au film after annealing at 650 °C, and after the annealing and following introducing Ag^{+} ions in glass and additional heat treatment at 600 °C.
